# Analysis of Stereotyped B-Cell Receptor Frequencies Among Portuguese De Novo-Diagnosed Chronic Lymphocytic Leukemia Patients (PAIS Study)

**DOI:** 10.3390/cancers17081316

**Published:** 2025-04-14

**Authors:** Daniela Alves, Gisela Ferreira, Joana Caldas, Mariana Fernandes, Cátia Gaspar, Mafalda Alpoim, Inês Carvalhais, Sara Duarte, Helena Silva, Ana Montalvão, Fernanda Vargas, Teresa Ribeiro, Ana António, Rita Coutinho, Francisca Miranda, Tânia Maia, Marta Gomes, José Carda, Sónia Matos, Rita Jaime, João Raposo

**Affiliations:** 1 Unidade Local de Saúde Santa Maria, E.P.E, 1649-035 Lisboa, Portugal; 2 Unidade Local de Saúde de Aveiro, 3810-501 Aveiro, Portugal; 3 Hospital Santo António dos Capuchos, Unidade Local de Saúde de São José, 1169-050 Lisboa, Portugal; 4 CUF Descobertas, 1998-018 Lisboa, Portugal; 5 Unidade Local de Saúde de Gaia/Espinho, E.P.E, 4434-502 Vila Nova de Gaia, Portugal; 6 Centro Hospitalar Universitário de São João, Unidade Local de Saúde de São João, E.P.E, 4200-319 Porto, Portugal; 7 Centro Hospitalar e Universitário de Coimbra, Unidade Local de Saúde de Coimbra, E.P.E, 3004-561 Coimbra, Portugal; 8 Hospital São Teotónio de Viseu, Unidade Local de Saúde de Viseu Dão-Lafões, E.P.E, 3504-509 Viseu, Portugal; 9 Unidade Local de Saúde do Baixo Alentejo, E.P.E, 7801-849 Beja, Portugal; 10 Hospital Garcia de Orta, Unidade Local Saúde de Almada-Seixal, E.P.E, 2805-267 Almada, Portugal; 11 Hospital de Braga, Unidade Local de Saúde de Braga, E.P.E, 4710-243 Braga, Portugal; 12 Hospital de São Bernardo, Unidade Local de Saúde da Arrábida, E.P.E, 4400-346 Vila Nova de Gaia, Portugal; 13 Hospital de Santo António, Unidade Local de Saúde de Santo António, E.P.E, 4050-342 Porto, Portugal; 14 Hospital de São Francisco Xavier, Unidade Local de Saúde de Lisboa Ocidental, E.P.E, 1449-005 Lisboa, Portugal; 15 Hospital Pedro Hispano, Unidade Local de Saúde de Matosinhos, E.P.E, 4464-513 Matosinhos, Portugal; 16 Hospital da Luz de Lisboa, 1500-650 Lisboa, Portugal; 17 GenoMed, Diagnósticos de Medicina Molecular, SA, 1649-028 Lisboa, Portugal; 18 Johnson & Johnson Innovative Medicine, 2740-262 Porto Salvo, Portugal

**Keywords:** B-cell receptor, B lymphocytes, BcR, chronic lymphocytic leukemia, CLL, IGHV, immunoglobulin heavy-chain variable region gene, leukemia

## Abstract

Background/Objectives: Chronic lymphocytic leukemia (CLL) exhibits a heterogeneous clinical course influenced by genetic factors, such as the mutational status of immunoglobulin variable regions (*IGHV*). Recently, B-cell receptor (BcR) stereotypes have shown promising prognostic value, potentially surpassing *IGHV* status. The PAIS study analyzed BcR stereotypes and *IGHV* mutations in newly diagnosed Portuguese CLL patients to assess prognostic characteristics and disease progression. Methods: This cross-sectional study included 463 adult patients from 15 Portuguese centers, recruited between November 2020 and September 2023. The median age at diagnosis was 70.4 years. The most common clinical stages were 0 (54%) and 1 (32.83%). Results: A total of 15 different BcR stereotypes were identified in the cohort studied. Subtype #1, associated with a poorer prognosis, was the most prevalent, observed in 3.90% of newly diagnosed Portuguese CLL patients. Considering the 19 major stereotypes that could be assigned by the ARResT subsets tool, most patients exhibited a heterogeneous BcR profile (90.14%). A total of 57.24% of patients had mutated *IGHV*. The concentration of β2-microglobulin was significantly lower in patients with mutated *IGHV* (2.6 mg/L vs. 3.6 mg/L, *p* < 0.001). Clinical stage, assessed by the RAI staging system, differed between subgroups, with a higher frequency of stage 0 in patients with mutated *IGHV* and stage 2 in unmutated patients (*p* = 0.009). Conclusions: The PAIS study highlighted the predominance of a heterogeneous BcR profile in Portuguese CLL patients. The higher percentage of patients with mutated *IGHV* at diagnosis supports prior findings. This study improves the characterization of the 10% of Portuguese CLL patients with major BcR stereotypes, offering healthcare providers better predictive power for disease progression and potentially impacting clinical decision making.

## Simple Summary

Chronic lymphocytic leukemia (CLL) is a type of blood cancer that progresses differently in each patient, influenced by genetic factors. One key factor is the *IGHV* gene, which helps predict disease progression. However, scientists have recently discovered that patterns in B-cell receptors (BcR stereotypes) may offer even better predictions. This study (PAIS) examined the BcR stereotypes and *IGHV* gene status in 463 recently diagnosed CLL patients in Portugal. The findings showed that most patients had a diverse BcR profile, but a small group had specific stereotypes linked to a worse prognosis. Additionally, over half of the patients had a mutated *IGHV* gene, which is generally linked to a more favorable outcome. By identifying these genetic patterns, this study helps doctors better predict disease progression, ultimately improving patient care and treatment decisions.

## 1. Introduction

Chronic lymphocytic leukemia (CLL) is the most prevalent type of leukemia among adults in Western countries [1]. Worldwide, the incidence of CLL rose between 1990 and 2019 [2]. In Portugal, the incidence rate was 5.22 per 100,000 inhabitants in 2014 [3]. This disease primarily affects older adults, with a median age at diagnosis of 70 years [4], and occurs slightly more frequently in men than in women [5]. CLL is characterized by the progressive proliferation and accumulation of mature, immuno-incompetent B lymphocytes in the peripheral blood, bone marrow, and secondary lymphoid tissues [6].

The immunophenotype of CLL is well characterized, and a major consensus identified CD19, CD5, CD23, CD20, CD79b, CD200, CD43 and surface light chains as essential markers for the diagnosis of this malignancy [7,8]. CLL staging systems are used to classify the disease based on its severity and extent. The two most commonly used staging systems are the Rai system [9] and the Binet system [10]. Both are used to define disease burden and treatment indication, based on standard laboratory findings and physical examination. In daily practice, both staging systems assist in categorizing patients based on disease risk.

The clinical progression of CLL is marked by considerable heterogeneity. While some patients experience minimal impact on survival, others face a more aggressive disease course marked by progression and complicated by infections and autoimmune disorders [11]. Selecting the appropriate treatment for each CLL patient demands a comprehensive assessment of disease characteristics, prior treatment history, patient preferences, and comorbidities [12]. However, several biomarkers are available to provide additional prognostic insights, including IG heavy-chain variable region (IGHV) genes mutational status, serum β2-microglobulin, and the presence of *del(17p)* and/or *TP53* mutations [13].

The B-cell receptor (BcR) is fundamental for CLL pathogenesis and is composed of immunoglobulin (IG) molecules plus CD79a/b subunits [14]. Immunogenetically, there are two molecular subgroups: those with unmutated *IGHV* genes (U-CLL, ≥98% identity with the germline) and those with mutated *IGHV* genes (M-CLL) [15]. U-CLL arises from B-cells that have not undergone the germinal center process, while M-CLL develops from B-cells that have emerged from the germinal center [16]. In addition, around 41% of patients have highly homologous amino acid sequences derived from almost identical IG rearrangements, known as stereotypes, which correspond to 13% of the 19 most common stereotypes and 28% of rare and/or novel stereotypes [17].

Over the past decade, advancements in our understanding of CLL biology have led to significant improvements in treatment approaches [18]. A critical factor in determining the clinical course of CLL is the disease’s genetic profile, with the mutational status of the *IGHV* gene emerging as a key prognostic marker that influences patient outcomes independently of clinical stage [19]. Patients with unmutated *IGHV* (U-CLL) generally experience more aggressive disease progression and shorter survival times compared to those with mutated *IGHV* (M-CLL) [19]. In early-stage CLL, unmutated *IGHV* status at diagnosis independently predicts a shorter time to first treatment [20], and *IGHV* gene somatic hypermutation (SHM) status remains a valuable prognostic tool across various outcome measures.

Beyond *IGHV* mutational status, BcR stereotypes provide an additional layer of prognostic precision, as patients within the same stereotyped subset exhibit highly consistent biological and clinical features, including shared antigen reactivity, genomic aberrations, and signaling characteristics [17,19]. These shared features persist even when *IGHV* mutational status varies among patients, highlighting the influence of antigen selection in CLL pathobiology [21]. Notably, specific stereotyped subsets have demonstrated clinical relevance beyond *IGHV* status alone. For instance, subsets #1 and #6, which are associated with U-CLL, display aggressive disease behavior, while subset #2, despite being observed in both M-CLL and U-CLL, is linked to poor response to chemoimmunotherapy. Additionally, subset #8 carries the highest risk for Richter transformation among all CLL cases (Table 1) [16,17,22]. The uniformity within these subsets suggests that BcR stereotypes serve as robust biomarkers for disease stratification, refining prognostic assessments and guiding therapeutic decision making more effectively than *IGHV* status alone.

In the coming years, the prevalence and mortality of CLL are expected to rise due to an increase in incidence among older populations [24]. Unravelling disease biomarkers and the development of new therapies in the era of precision medicine, targeting the individual risk profile of each patient are critical to potentiate the possible clinical benefit [18]. Likewise, clinically relevant prognostic biomarkers, such as *IGHV* mutation status, are fundamental for CLL patient counseling, treatment tailoring and prediction of treatment response. The most recent guidelines support *IGHV* mutation analysis as an additional test before starting treatment [13], and recommendations on the minimal technical requirements for a reliable and reproducible analysis of the rearranged *IGHV* sequences have been issued [19]. In this context, given the clinical–biological associations observed in specific subsets with stereotyped BcR, it is conceivable that future therapeutic decisions should be informed not only by the mutational status of *IGHV* genes but also by the unique characteristics of individual CDR3 regions [25,26].

In the PAIS study, we have analyzed the frequency of BcR stereotypes and *IGHV* mutational status in Portuguese patients with newly diagnosed CLL and correlated these factors with the patient’s demographic and clinical characteristics.

## 2. Materials and Methods

### 2.1. Study Design and Patients

The PAIS study is a cross-sectional, multicenter, non-interventional study conducted in 15 Portuguese sites (CUF Descobertas, Hospital de Braga/Unidade Local de Saúde (ULS) de Braga, Hospital de Santa Maria/ULS de Santa Maria, Hospital de Santo António/ULS de Santo António, Hospital de São Bernardo/ULS da Arrábida, Hospital de São Francisco Xavier/ULS de Lisboa Ocidental, Hospital Garcia de Orta/ULS de Almada-Seixal, Hospital José Joaquim Fernandes/ULS do Baixo Alentejo, Hospital Pedro Hispano/ULS de Matosinhos, Hospital S. António dos Capuchos/ULS de São José, Hospital São Teotónio de Viseu/ULS de Viseu Dão-Lafões, Centro Hospitalar do Baixo Vouga/ULS da Região de Aveiro, Hospital Universitário de Coimbra/ULS de Coimbra, Centro Hospitalar de Vila Nova de Gaia-Espinho/ULS Gaia/Espinho, and Hospital de São João/ULS de São João). Study enrollment took place from November 2020 to September 2023, and the main inclusion criteria were as follows: patients aged ≥18 years born in Portugal, and a confirmed de novo diagnosis of CLL (under International Workshop on Chronic Lymphocytic Leukemia (iwCLL) criteria [13]). De novo-diagnosed CLL patients were defined as patients newly diagnosed with CLL, who have been informed about the diagnosis by their treating physician up to 3 months before inclusion, and who have not started active CLL therapy. Patients who have lived outside Portugal for more than 5 years, within the 10 years prior to the CLL diagnosis, were excluded from the study.

Demographic and clinical data available in patient’s medical records per clinical practice were collected within this study. Additionally, peripheral blood samples were collected during routine CLL assessment, and a fraction was used for determination of *IGHV* mutational status and for analysis of the BcR stereotype. Mutated *IGHV* was defined as mutation rates of >2% difference from germline *IGHV* gene, as reported by the international ImMunoGeneTics information system—IMGT, with a 98% homology cutoff. Both analyses were conducted in a Portuguese certified laboratory—GenoMed, Diagnósticos de Medicina Molecular, S.A.

The study was conducted according to the Declaration of Helsinki and principles of good clinical practice (GCPs), with the approval of the Independent Ethics Committee at each participating site. All patients provided written informed consent to participate in the study. This report followed the “Strengthening the Reporting of Observational Studies in Epidemiology” (STROBE) guidelines for reporting observational studies [27].

### 2.2. Study Endpoints

The primary endpoint was defined as the frequency of BcR stereotypes among Portuguese patients with newly diagnosed CLL, determined through analysis in a certified laboratory. The secondary endpoints included determining the *IGHV* mutational status of “de novo”-diagnosed CLL patients and assessing demographic and clinical characteristics of patients according to BcR stereotypes and respective *IGHV* mutational status. Demographic and clinical data available in patient’s medical records per clinical practice were collected within this study or collected at the study’s single visit. The following demographic data were collected: age at diagnosis, gender, birthplace (district), height (cm), weight (kg). Detailed medical history was also collected, such as comorbidities and respective date of diagnosis; CLL characteristics: date of diagnosis and date of communication to the patient if different; clinical stage (Rai staging system—0 to IV), *del(17p)* (yes/no/not available), *TP53* mutations (yes/no/not available), β2-microglobulin concentration (mg/L/not available), BcR characterization (stereotype, *IGHV* mutational status (mutated/unmutated/not determined), and homology percentage of *IGHV* mutational status (laboratory testing using leader primers and 98% as the homology cutoff value, as previously described [19]) were collected. Unavailable data were considered missing.

### 2.3. Statistical Analysis

Continuous variables were presented as mean values ± standard deviations or median values (Q1 (first quartile) and Q3 (third quartile). For independent samples, parametric (*t*-test) or non-parametric (Mann–Whitney) statistical tests were performed depending on the sample distribution (normality test). For paired samples, parametric (*t*-test) or non-parametric (Wilcoxon) statistical tests were performed depending on the sample distribution. Categorical variables were described using absolute and relative frequencies (*n*, %). 95% confidence intervals were calculated. To compare proportions and/or frequency distribution, chi-square tests (or Fisher’s exact test, if appropriate) were performed. The analysis of demographic and clinical characteristics, and CLL characteristics for each BcR stereotype, was carried out for the BcR stereotypes when *n* ≥ 20 patients. Statistical comparisons were performed at a significance level of 0.05. Data were analyzed using software SAS, version 9.4, under Enterprise Guide interface version 8.3.

## 3. Results

### 3.1. Study Population and Baseline Characteristics

A total of 472 patients were enrolled in the study. Seven patients were excluded for not fulfilling the eligibility criteria and two patients were excluded due to incompleteness of the information collected in the BcR characteristics form. This resulted in 463 eligible patients for analysis.

The demographic and clinical characteristics of patients are summarized in Table 1 and Table 2. The median age of patients was 72 (Q1–Q3: 62.0–79.0) years, and the percentage of male patients was 59.8% (Q1–Q3: 59.14–60.51) (Table 2). Birthplace details of the enrolled patients are shown in Table 1. Patients were representative of all Portuguese districts, except Viana do Castelo (*n* = 0). The most represented regions were Aveiro and Lisbon, with 18.6% and 20.9% of patients, respectively (Table 2). The median body mass index (BMI) of the cohort was 26.6 (Table 2) and the most frequent comorbidity was hypertension, which was present in 61.7% of patients (*n* = 282) (Table 3). Regarding the CLL clinical stage at baseline, most patients were in Stage 0 (54%; *n* = 250) and Stage I (32.8%; *n* = 152), according to the Rai staging system (Table 3). Information on the *del(17p)* mutation was available for only two patients (0.4% of the cohort), while three patients (0.7%) carried the *TP53* mutation (Table 3). The majority of patients did not undergo these analyses at the time of recruitment, as they were in an early asymptomatic stage and did not require treatment. Additionally, these tests are considered optional for patients who do not meet the criteria for treatment initiation. The median β2-microglobulin concentration was 2.4 mg/L (CI: 2.0–3.4) (Table 3).

### 3.2. BcR Stereotypes Frequency Among Portuguese Patients with De Novo CLL Diagnosis

A total of 15 distinct BcR subsets were identified among the Portuguese study cohort. The most prevalent subset, subset #1, was observed on 3.90% of the Portuguese patients with de novo CLL diagnosis (Table 4). Less frequent subsets included subsets #6 and #8, each with a frequency of 0.9%; subsets #4 and #77, each with a frequency of 0.7%; and subsets #2 and #59, each with a frequency of 0.5%. The subsets #3, # 5, #14, #16, #28A, #64B, #99, and #201 were each detected at a frequency of 0.2% (Table 4). Notably, 90.14% of the Portuguese patients exhibited a heterogeneous BcR profile (Table 4). The distribution of the BCR stereotypes according to the birthplace is described in Table S1.

### 3.3. IGHV Mutational Profile Among Portuguese Patients with De Novo CLL Diagnosis

Regarding the determination of the *IGHV* mutational status of “de novo”-diagnosed CLL patients (Table 5), 57.24% of the patients held a mutated *IGHV*, with 93.8% and 94.1% median homology rates using leader primers. It was observed that 32 patients in the M-*IGHV* group and 27 patients in the U-*IGHV* group had two simultaneous productive rearrangements.

### 3.4. Demographic Characterization of Newly Diagnosed Portuguese CLL Patients According to the BcR Profile (Heterogeneous)

Considering the BcR profile, complete assessment of the demographic and clinical characteristics was conducted exclusively on the subpopulation with a heterogeneous BcR stereotype (*n* = 393), as this was the sole subgroup with a sample size ≥ 20 patients (Table 6). The median age of patients in this subpopulation was 71.0 (Q1–Q3: 62.0–79.0) years, with predominance of the male gender (60.6%) and a median BMI of 26.8 (Q1–Q3: 24.2–29.8) (Table 6).

### 3.5. Clinical Characterization of Newly Diagnosed Portuguese CLL Patients According to the BcR Profile (Heterogeneous)

In the subgroup of patients with a heterogeneous BcR profile, the most observed comorbidities were hypertension (63.4%), diabetes (24.1%) and dyslipidemia (23.1%) (Table 7). According to the clinical Rai staging system, 53.2% patients were in Stage 0, 33.3% in Stage I, 7.9% in Stage II, 2.5% in Stage III and 3.1% in Stage IV (Table 7). Mutation analysis revealed that 2.3% of 88 patients with a heterogeneous BcR profile exhibited *del(17p)*, and 13.3% of 15 patients analyzed had *TP53* gene mutations (Table 7). Notably, most patients did not undergo these analyses, as they are only conducted when treatment criteria are met. The median B2-microglobulin concentration of this subset of patients was 2.4 (Q1–Q3 2.0–3.4) mg/L (Table 7).

### 3.6. Demographic Characterization of Newly Diagnosed Portuguese CLL Patients According to the IGHV Mutational Status (Mutated vs. Unmutated)

Age at diagnosis, gender, and BMI of patients with mutated or unmutated *IGHV* were not significantly different. The median age was 71.0 years (Q1–Q3: 61.0–80.0) in the mutated subpopulation and 72.0 years (Q1–Q3: 64.0–78.0) in the unmutated subpopulation (Table 8). Males comprised 58.5% of the mutated group and 60.8% of the unmutated group (Table 8). Patients with unmutated *IGHV* had a median BMI of 26.8 (Q1–Q3: 24.2–29.7), while those with mutated *IGHV* had a median BMI of 26.1 (Q1–Q3: 24.1–29.6) (Table 8). Although sample sizes per district were limited, the distribution of *IGHV* mutational status by birthplace suggests that patients with mutated *IGHV* are present across most regions, except in Évora and Santarém. In Évora, six of seven patients (85.7%) had unmutated *IGHV*, while only one patient (14.3%) had mutated *IGHV*. In Santarém, 19 of 33 patients (57.6%) had unmutated *IGHV*, and 14 patients (42.4%) had mutated *IGHV* (see Table 8 and Supplementary Table S2).

### 3.7. Clinical Characterization of Newly Diagnosed Portuguese CLL Patients According to the IGHV Mutational Status (Mutated vs. Unmutated)

The clinical characteristics of patients, stratified by *IGHV* mutational status, are summarized in Table 9. There was a statistically significant difference between the two groups in terms of comorbidities: cardiovascular diseases (other than hypertension and atrial fibrillation) were more prevalent in patients with mutated *IGHV* (*p* < 0.001) (Table 9).

The analysis of CLL characteristics based on *IGHV* mutational status revealed significant differences in clinical stage between the groups. Mutated *IGHV* patients had a higher frequency of stage 0 disease (58.5% compared to 44.4% in the unmutated group) (Table 9). Conversely, unmutated *IGHV* patients had higher frequencies of stage II, III, and IV disease (12.3%, 4.1%, and 4.1%, respectively, versus 5.7%, 1.5%, and 2.3% in the mutated group) (*p* = 0.009) (Table 9). Moreover, β2-microglobulin concentration differed significantly between the two patient subsets, with a median concentration of 2.3 mg/L (Q1–Q3: 1.9–2.9) in the mutated *IGHV* group compared to 2.7 mg/L (Q1–Q3: 2.1–4.3) in the unmutated group (*p* < 0.001) (Table 9).

## 4. Discussion

Herein, we present the final results of the PAIS study. To the best of our knowledge, this is the first multicentric study in Portugal to collect real-world evidence on newly diagnosed chronic lymphocytic leukemia (CLL) patients. This study aimed to determine the frequency of B-cell receptor (BcR) stereotypes and *IGHV* mutational status, while also evaluating the demographic and clinical characteristics of these patients. Given the heterogeneity of clinical practices across countries, this research holds significant relevance for tailoring CLL treatment, predicting therapeutic response, and providing insights into the national clinical experience.

The study determined that 90.14% of Portuguese CLL patients exhibited a heterogeneous (non-stereotyped) BcR profile. However, this finding may be influenced by both sample size and technical limitations, as the classification of heterogeneous profile can include minor stereotyped subsets with a frequency below 0.2% not identified by the ARResT subsets tool, potentially leading to an overestimation of the true prevalence of heterogeneous BcR subtypes. Despite this consideration, the observed proportion remains significantly higher than the 60–78% range reported in previous studies from Europe and the United States [17,28] and even in Asia (66–82%) [25,29,30,31]. This suggests a particularly heterogeneous nature of CLL in Portuguese patients, potentially indicating different antigenic exposures or other environmental factors unique to this population. However, these results must be interpreted with caution due to the sample size.

Among the approximately 10% of patients with stereotyped receptors, 15 distinct subsets were identified, with subset #1 being the most prevalent (3.9%), followed by subsets #6 and #8 (0.9% each). The prevalence of subset #1 aligns with previous findings from a German CLL cohort [22], and a large cohort from Europe, the United States, and Asia, where it was identified as the second most common subset, with a prevalence of 2.1% [17]. Subset #1 is associated with an aggressive disease course [32], characterized by a poor prognosis with respect to treatment-free interval [22] and a shorter time to first treatment [33]. Similarly, subset #8, known for its prevalence among Taiwanese CLL patients and its association with poorer clinical outcomes [27], was also present in our cohort as the second most common subset (0.9%). Particularly, subset #8 exhibits the highest risk for Richter transformation among all CLL subsets [15]. Subset #6, another well-characterized clinically aggressive CLL subgroup [15], was equally prevalent in the Portuguese cohort, sharing the position of the second most common subset with subset #8 (0.9%). Subsets #4 and #77 were the third most represented (0.7%). CLL patients assigned to subset #4 display a particularly indolent disease course [34], with an early age at disease onset [26,33,35]. Interestingly, subset #77 has been described as more common in Asian populations [29]. Subset #2, although the most frequent major subset in Western CLL cohorts [25], was present in only 0.5% of our dataset. These findings highlight the unique composition of stereotyped subsets in Portuguese CLL patients and suggest inherent differences in chronic antigenic interactions during CLL pathogenesis between populations, however these results may be influenced by sample size and recruitment bias.

Many studies that determine *IGHV* mutation status at the time of treatment initiation may inadvertently introduce selection bias. Patients requiring early treatment due to aggressive disease (often with unmutated *IGHV*) are included, while those with more indolent disease (often with mutated *IGHV*) may be excluded [15,16]. In the present study, *IGHV* mutation status was assessed at the time of diagnosis, independent of treatment initiation, allowing for a more comprehensive characterization of the patient population. *IGHV* mutational analysis revealed a higher proportion of mutated *IGHV* cases (57.2%), consistent with the prevalence reported in newly diagnosed, asymptomatic CLL patients in other studies [17,25,29]. This finding reinforces the notion that mutated *IGHV* is more common at the initial diagnosis, while unmutated *IGHV* genes are more prevalent among progressive (50–60%) and relapsed/refractory (70–80%) CLL patients [36]. Additionally, *IGHV*-mutated CLL is also associated with earlier stages of disease and good prognosis [19,20]. This is in line with the findings in the Portuguese cohort, where the clinical stage at diagnosis varied between mutated and unmutated patients, with a higher proportion of mutated cases presenting at stage 0 (58.5%) compared to unmutated cases (44.4%). Furthermore, elevated β2-microglobulin levels in the unmutated group indicate a more aggressive disease phenotype, as high β2-microglobulin is correlated with advanced disease stages and poor clinical outcomes in CLL [37]. Therefore, patients with unmutated *IGHV* in our cohort may require closer monitoring and early intervention strategies due to the association with higher β2-microglobulin levels and more advanced clinical stages of the disease. Also, dyslipidemia was significantly higher in patients with unmutated *IGHV,* which, considering the potential role of cholesterol in disease progression [38], highlights the relevance of monitoring and managing cholesterol levels as part of the routine management of patients with CLL.

Several limitations of this study should be acknowledged. Although the overall sample size was relatively large for a study conducted in Portugal (*n* = 463), some clinical data were incomplete due to the retrospective nature of chart-based data collection in routine clinical practice. Additionally, the number of patients per BcR stereotyped group was small, limiting our ability to explore clinical or demographic patterns within individual subsets. While we recruited patients from across the entire country, regional representation was uneven, which precludes definitive conclusions regarding the geographic distribution of *IGHV* mutational status or BcR stereotypes. Moreover, the study design was cross-sectional, and as such, it did not include longitudinal follow-up data, hindering the ability to assess clinical outcomes, such as disease progression or treatment response—though follow-up is currently underway and could be addressed in future analyses. Finally, data on *TP53* mutational status were not collected for the majority of patients. This was a deliberate methodological choice, as the primary focus of the present study was the immunogenetic profiling of the cohort. This study is not intended to influence local clinical practice and the tests in accordance with Portuguese clinical practice are not performed for the majority of patients who do not meet the criteria for treatment initiation, as outline by the iwCLL guidelines. Nonetheless, we acknowledge the prognostic significance of *TP53* abnormalities and their importance in future molecular risk stratification efforts.

The results of this study can be generalized to the adult population of newly diagnosed CLL patients in Portugal. The sample size was relatively large compared to similar studies in countries with larger populations, and demographic characteristics were comparable to previous epidemiological studies of CLL in Portugal [3,30,31]. However, the unique demographic, clinical, and genetic characteristics observed in the Portuguese population suggest that these findings may not be directly applicable to other populations without further validation. These observations underscore the need for continued research, including prospective follow-up and expanded molecular profiling, to deepen our understanding of disease heterogeneity and progression in this population.

## 5. Future Directions

The findings of the PAIS study lay the groundwork for several important avenues of future research. While the current analysis provides a robust baseline characterization of the immunogenetic landscape in Portuguese CLL patients, the follow-up of this cohort is ongoing and will allow for a longitudinal assessment of clinical outcomes. This includes evaluation of time to first treatment, progression-free and overall survival, and risk of transformation—particularly in relation to BcR stereotypy and *IGHV* mutational status. Such longitudinal data will be instrumental in validating the prognostic relevance of immunogenetic markers in the Portuguese population and guiding their use in clinical decision making.

In future phases of the study, we also aim to expand the molecular profiling of patients to include genomic alterations with well-established prognostic value, such as *TP53* mutations and *del(17p)*. Integrating these genomic markers with BcR and *IGHV* profiles may reveal synergistic patterns that refine risk stratification algorithms and support more personalized treatment approaches.

To address current geographic imbalances in recruitment, future efforts will also focus on increasing enrollment from underrepresented regions, including rural and lower-population areas. This will improve the representativeness of the dataset and may enable the exploration of environmental or regional factors that could contribute to differences in CLL immunogenetic profiles across Portugal.

Lastly, the PAIS database presents an opportunity to investigate the interplay between CLL biology and patient comorbidities. Initial findings of higher dyslipidemia rates among unmutated *IGHV* cases raise the possibility of metabolic factors influencing disease course. Exploring such associations in greater depth could generate novel hypotheses about the systemic contributors to CLL progression and help identify additional modifiable risk factors in disease management.

## 6. Conclusions

In conclusion, the PAIS study provided valuable insights into the immunogenetic landscape of CLL among Portuguese patients, revealing a predominantly heterogeneous BcR profile. This finding suggests a complex and varied pattern of antigenic exposures in this population, potentially influenced by regional environmental or genetic factors. The higher prevalence of BcR heterogeneity compared to the previous literature underscores the need for localized studies to capture the full spectrum of disease variability, which may not be fully represented in broader, more generalized datasets.

Moreover, the study’s observation of a significant proportion of patients with mutated *IGHV* genes aligns with established data, reinforcing the importance of *IGHV* mutation status as a critical prognostic marker. This consistency with global findings validates the robustness of the study’s methodology and highlights the relevance of these molecular markers in the Portuguese clinical context.

Importantly, although only approximately 10% of patients exhibited stereotyped BcR subsets, the subset distribution differed from that of international cohorts, suggesting potential population-specific patterns of antigenic drive. The identification of aggressive subsets such as #1, #6, and #8 in the Portuguese cohort highlights the relevance of stereotype-based profiling for identifying high-risk individuals and refining clinical risk stratification.

This study also established, for the first time in Portugal, a large nationwide database encompassing epidemiological, clinical, immunogenetic, and comorbidity data from newly diagnosed CLL patients, providing a valuable foundation for future research.

However, several limitations should be acknowledged, including regional recruitment imbalances, the lack of *TP53* mutational data, and the absence of follow-up outcomes at this stage. These reflect the cross-sectional nature of the current analysis, and the predefined study focus on immunogenetic features. Nonetheless, the overall sample size is considerable for a single-country study and broadly representative of the national CLL population.

The ongoing follow-up of this cohort will enable future longitudinal analyses, including disease progression, treatment response, and survival outcomes. Furthermore, the planned integration of genomic markers such as *TP53* mutations and *del(17p)*, as well as expanded recruitment from underrepresented regions, will enhance the translational potential of the dataset. Additional efforts to investigate associations between CLL immunogenetics and comorbid conditions, such as dyslipidemia, may also offer novel insights into systemic factors modulating disease course.

The insights gained from the PAIS study, therefore, hold the potential to influence clinical decision making, ultimately contributing to more personalized patient care and improved outcomes for individuals affected by CLL in Portugal and other comparable clinical or demographic settings.

## Figures and Tables

**Table 1:** Biological and clinical characteristics of the major stereotyped subsets in CLL [14,23].

BcR Subtypes	*IGHV* Mutational Status	Signaling Properties	Genetic Alterations	Clinical Features
#1	Unmutated	Functional	Deletion of 11q, 17p, *NOTCH1*, *NFKBIE* mutations	Aggressive (median TTFT of 1.6 yrs)
#2	Mutated and Unmutated	Functional	Deletion of 11q and 13q, *SF3B1* mutations	Aggressive (median TTFT of 1.9 yrs)
#4	Mutated	Anergic BCRs	Deletion of 13q	Indolent (median TTFT of 11 yrs)
#8	Unmutated	Functional	Trisomy 12, *NOTCH1* mutations	Aggressive (median TTFT of 1.5 yrs), increased risk of Richter’s transformation

TTFT, time to first treatment.

**Table 2:** Baseline demographic characteristics of overall patient population.

Baseline Demographic Characteristics	Overall (*n* = 463) *n* (%)
Age at diagnosis	
Median (Q1–Q3)	72 (62.0–79.0)
Male sex	277 (59.8)
BMI	
Median (Q1–Q3)	26.6 (24.2–29.7)
Missing (*n*)	98
Birthplace	
Aveiro	86 (18.57)
Beja	26 (5.62)
Braga	23 (4.97)
Bragança	6 (1.30)
Castelo Branco	10 (2.16)
Coimbra	20 (4.32)
Évora	7 (1.51)
Faro	11 (2.38)
Guarda	10 (2.16)
Leiria	18 (3.89)
Lisboa	97 (20.95)
Portalegre	8 (1.73)
Porto	41 (8.86)
Santarém	34 (7.34)
Setúbal	16 (3.46)
Viana do Castelo	0 (0.00)
Vila Real	11 (2.38)
Viseu	37 (7.99)
Açores	1 (0.22)
Madeira	1 (0.22)
Missing (*n*)	0

BMI, body mass index; Q1, first quartile; Q3, third quartile. Values are represented as absolute numbers and % unless otherwise specified.

**Table 3:** Baseline clinical characteristics of overall patient population.

Baseline Clinical Characteristics	Overall (*n* = 463) *n* (%)
Comorbidities	
Hypertension	282 (61.7)
Missing (*n*)	6
Atrial Fibrillation	48 (10.5)
Missing (*n*)	8
Other cardiovascular disease	95 (20.8)
Missing (*n*)	7
Dyslipidemia	22 (23.2)
Missing (*n*)	0
Diabetes	104 (23.1)
Missing (*n*)	13
Psychiatric disease	30 (6.6)
Missing (*n*)	7
Respiratory disease	46 (10.1)
Missing (*n*)	6
Renal disease	28 (6.6)
Missing (*n*)	40
Neurological disease	36 (7.8)
Missing (*n*)	4
Rai staging system	
Stage 0	250 (54.0)
Stage I	152 (32.8)
Stage II	37 (8.0)
Stage III	11 (2.4)
Stage IV	13 (2.8)
Missing (*n*)	0
Mutational status (*del(17p)/TP53*)	
*del(17p)*	2 (0.4)
Missing (*n*)	365
*TP53* mutations	3 (0.7)
Missing (*n*)	445
β2-microglobulin concentration (mg/L)	
Median (Q1–Q3)	2.4 (2.0–3.4)
Missing (*n*)	199

BMI, body mass index; Q1, first quartile; Q3, third quartile. Values are represented as absolute numbers and % unless otherwise specified.

**Table 4:** BcR characterization of the Portuguese patients with de novo CLL diagnosis.

BcR Subtypes	Overall (*n* = 463) *n* (%)
#1	17 (3.9)
#2	2 (0.5)
#3	1 (0.2)
#4	3 (0.7)
#5	1 (0.2)
#6	4 (0.9)
#7H	0 (0.0)
#8	4 (0.9)
#12	0 (0.0)
#14	1 (0.2)
#16	1 (0.2)
#28A	1 (0.2)
#31	0 (0.0)
#59	2 (0.5)
#64B	1 (0.2)
#77	3 (0.7)
#99	1 (0.2)
#201	1 (0.2)
#202	0 (0.0)
Other subset	0 (0.0)
Heterogeneous *	393 (90.1)
Missing (*n*)	27

* Can include stereotypes with a frequency ≤ 0.2% not identified by the ARResT subsets tool.

**Table 5:** *IGHV* mutational status.

*IGHV* Mutational Status	Overall (*n* = 463)
Mutated (*n*, %)	265 (57.2)
Homology 1 (*n*)	265
Median (% (Q1–Q3))	93.8 (91.3–95.5)
Missing (*n*)	0
Homology 2 (*n*)	32
Median (% (Q1–Q3))	94.1 (90.5–96.5)
Missing (*n*)	233
Unmutated (*n*, %)	171 (36.9)
Homology 1 (*n*)	171
Median (% (Q1–Q3))	100.0 (100.0–100.0)
Missing (*n*)	0
Homology 2 (*n*)	27
Median (% (Q1–Q3))	100.0 (96.4–100.0)
Missing (*n*)	144

Q1, first quartile; Q3, third quartile.

**Table 6:** Demographic characteristics of newly diagnosed Portuguese CLL patients with a heterogeneous BcR profile.

Demographic Characteristics	Overall (*n* = 463) *n* (%)	Heterogeneous BcR (*n* = 393) *n* (%)
Age at diagnosis		
Median (Q1–Q3)	72 (62.0–79.0)	71.0 (62.0–79.0)
Male sex	277 (59.8)	238 (60.6)
Missing (*n*)	0	0
BMI		
Median (Q1–Q3)	26.6 (24.2–29.7)	26.8 (24.2–29.8)
Missing (*n*)	98	82

Q1, first quartile; Q3, third quartile.

**Table 7:** Clinical characteristics of newly diagnosed Portuguese CLL patients with a heterogeneous BcR profile.

Clinical Characteristics	Overall (*n* = 463) *n* (%)	Heterogeneous BcR (*n* = 393) *n* (%)
Comorbidities *		
Hypertension	282 (61.7)	246 (63.4)
Missing (*n*)	6	5
Atrial Fibrillation	48 (10.5)	41 (10.6)
Missing (*n*)	8	7
Other CVD	95 (20.8)	78 (20.2)
Missing (*n*)	7	6
Dyslipidemia	22 (23.2)	18 (23.1)
Diabetes	104 (23.1)	92 (24.1)
Missing (*n*)	13	11
Psychiatric disease	30 (6.6)	26 (6.7)
Missing (*n*)	7	6
Respiratory disease	46 (10.1)	43 (11.1)
Missing (*n*)	6	5
Renal disease	28 (6.6)	23 (6.4)
Missing (*n*)	40	36
Neurological disease	36 (7.8)	30 (7.71)
Missing (*n*)	4	4
Rai staging system		
Stage 0	250 (54.0)	209 (53.2)
Stage I	152 (32.8)	131 (33.3)
Stage II	37 (8.0)	31 (7.9)
Stage III	11 (2.4)	10 (2.5)
Stage IV	13 (2.8)	12 (3.1)
Missing (*n*)	0	0
Mutational status (*del(17p)/TP53*)		
*del(17p)*	2 (0.4)	2 (2.3)
Missing (*n*)	365	305
*TP53* mutations	3 (0.7)	2 (13.3)
Missing (*n*)	445	378
β2-microglobulin (mg/L)		
Median (Q1–Q3)	2.4 (2.0–3.4)	2.4 (2.0–3.4)
Missing (*n*)	199	162

* Present in at least 4 patients.

**Table 8:** Demographic characteristics of newly diagnosed Portuguese CLL patients with mutated and unmutated *IGHV* mutational profile.

Demographic Characteristics	Overall (*n* = 436) *n* (%)	Mutated *IGHV* (*n* = 265) *n* (%)	Unmutated *IGHV* (*n* = 171) *n* (%)	*p*-Value (Mutated vs. Unmutated *IGHV*)
Age at diagnosis				0.381 (a)
Median (Q1–Q3)	71.0 (62.0–79.0)	71.0 (61.0–80.0)	72.0 (64.0–78.0)	
Male sex	259 (59.4)	155 (58.5)	104 (60.8)	0.629 (b)
Missing (*n*)	0	0	0	
BMI				0.152 (a)
Median (Q1–Q3)	26.5 (24.2–29.6)	26.8 (24.2–29.7)	26.1 (24.1–29.6)	
Missing (*n*)	90	61	29	
Birthplace				<0.001 (c)
Aveiro	78 (17.9)	54 (20.4)	24 (14.0)	
Beja	22 (5.1)	13 (4.9)	9 (5.3)	
Braga	23 (5.3)	14 (5.3)	9 (5.3)	
Bragança	6 (1.4)	3 (1.1)	3 (1.8)	
Castelo Branco	10 (2.3)	6 (2.3)	4 (2.3)	
Coimbra	20 (4.6)	15 (5.7)	5 (2.9)	
Évora	7 (1.6)	1 (0.4)	6 (3.5)	
Faro	10 (2.3)	5 (1.9)	5 (2.9)	
Guarda	9 (2.1)	6 (2.3)	3 (1.8)	
Leiria	17 (3.9)	11 (4.2)	6 (3.5)	
Lisbon	90 (20.6)	52 (19.6)	38 (22.2)	
Portalegre	8 (1.8)	6 (2.3)	2 (1.2)	
Oporto	39 (8.9)	25 (9.4)	14 (8.2)	
Santarém	33 (7.6)	14 (5.3)	19 (11.1)	
Setúbal	15 (3.4)	10 (3.8)	5 (2.9)	
Viana do Castelo	0 (0.0)	0 (0.0)	0 (0.0)	
Vila Real	11 (2.5)	7 (2.6)	4 (2.3)	
Viseu	36 (8.3)	21 (7.9)	15 (8.8)	
Azores	1 (0.2)	1 (0.4)	0 (0.0)	
Madeira	1 (0.2)	1 (0.4)	0 (0.0)	
Missing (*n*)	0	0	0	

(a): Mann–Whitney’s U test. (b): Chi-square test. (c): Fisher test.

**Table 9:** Clinical characteristics of newly diagnosed Portuguese CLL patients with mutated and unmutated *IGHV* mutational profile.

Clinical Characteristics	Overall (*n* = 436) *n* (%)	Mutated *IGHV* (*n* = 265) *n* (%)	Unmutated *IGHV* (*n* = 171) *n* (%)	*p*-Value (Mutated vs. Unmutated *IGHV*)
Comorbidities *				
Hypertension	272 (63.1)	166 (63.6)	106 (62.4)	0.793 (a)
Missing (*n*)	5	4	1	
Atrial Fibrillation	48 (11.2)	26 (10.0)	22 (13.0)	0.333 (a)
Missing (*n*)	7	5	2	
Other CVD	90 (20.9)	55 (21.1)	35 (20.7)	<0.001 (b)
Missing (*n*)	6	4	2	
Dyslipidemia	21 (23.3)	12 (21.8)	9 (25.7)	
Diabetes	99 (23.4)	60 (23.4)	39 (23.2)	0.958 (a)
Missing (*n*)	12	9	3	
Psychiatric disease	29 (6.7)	13 (5.0)	16 (9.5)	0.070 (a)
Missing (*n*)	6	4	2	
Respiratory disease	46 (10.7)	27 (10.3)	19 (11.2)	0.785 (a)
Missing (*n*)	5	4	1	
Renal disease	27 (6.8)	13 (5.4)	14 (9.0)	0.163 (a)
Missing (*n*)	38	23	15	
Neurological disease	33 (7.6)	22 (8.4)	11 (6.4)	0.445 (a)
Missing (*n*)	4	4	0	
Rai staging system				0.009 (a)
Stage 0	231 (53.0)	155 (58.5)	76 (44.4)	
Stage I	145 (33.3)	85 (32.1)	60 (35.1)	
Stage II	36 (8.3)	15 (5.7)	21 (12.3)	
Stage III	11 (2.5)	4 (1.5)	7 (4.1)	
Stage IV	11 (2.5)	6 (2.3)	7 (4.1)	
Missing (*n*)	0	0	0	
Mutational status (*del(17p)/TP53*)				
*del(17p)*	2 (2.2)	0 (0.0)	2 (5.0)	0.186 (b)
Missing (*n*)	344	213	131	
*TP53* mutations	3 (18.7)	1 (20.0)	2 (18.2)	1.000 (b)
Missing (*n*)	420	260	160	
β2-microglobulin (mg/L)				<0.001 (c)
Median (Q1–Q3)	2.4 (2.0–3.4)	2.3 (1.9–2.9)	2.7 (2.1–4.3)	
Missing (*n*)	186	111	75	

* Present in at least 4 patients. (a): Chi-square test. (b): Fisher test. (c): Mann–Whitney’s U test.

## Data Availability

The datasets presented in this article are not readily available because they are part of an ongoing study. Requests to access the datasets should be directed to rjaime3@its.jnj.com.

## Supplementary Materials

The following supporting information can be downloaded at: https://www.mdpi.com/article/10.3390/cancers17081316/s1, Table S1: Distribution of the BCR stereotypes of newly diagnosed Portuguese CLL patients according to birthplace (n (%)). Table S2: Distribution of the IGHV mutational status of newly diagnosed Portuguese CLL patients according to birthplace.

## References

[1] Byrd J.C., Flynn J.M., Niederhuber J.E., Armitage J.O., Doroshow J.H., Kastan M.B., Tepper J.E. (2014). 102—Chronic Lymphocytic Leukemia. Abeloff’s Clinical Oncology.

[2] Ou Y., Long Y., Ji L., Zhan Y., Qiao T., Wang X., Chen H., Cheng Y. (2022). Trends in Disease Burden of Chronic Lymphocytic Leukemia at the Global, Regional, and National Levels from 1990 to 2019, and Projections Until 2030: A Population-Based Epidemiologic Study. Front. Oncol..

[3] Cardoso Borges F., Ramos A., Lourenço A., Gomes da Silva M., Miranda A. (2021). Detailing the epidemiological and clinical characteristics of chronic lymphocytic leukaemia in Portugal-Results from a population-based cancer registry cohort study. PLoS ONE.

[4] (2021). The Surveillance, Epidemiology, and End Results (SEER) Program of the National Cancer Institute. Cancer Stat Facts: Leukemia—Chronic Lymphocytic Leukemia (CLL). https://seer.cancer.gov/statfacts/html/clyl.html.

[5] Siegel R.L., Miller K.D., Jemal A. (2020). Cancer statistics, 2020. CA Cancer J. Clin..

[6] Alaggio R., Amador C., Anagnostopoulos I., Attygalle A.D., Araujo I.B.O., Berti E., Bhagat G., Borges A.M., Boyer D., Calaminici M. (2022). The 5th edition of the World Health Organization Classification of Haematolymphoid Tumours: Lymphoid Neoplasms. Leukemia.

[7] Rawstron A.C., Kreuzer K.A., Soosapilla A., Spacek M., Stehlikova O., Gambell P., McIver-Brown N., Villamor N., Psarra K., Arroz M. (2018). Reproducible diagnosis of chronic lymphocytic leukemia by flow cytometry: An European Research Initiative on CLL (ERIC) & European Society for Clinical Cell Analysis (ESCCA) Harmonisation project. Cytom. B Clin. Cytom..

[8] van Dongen J.J.M., Lhermitte L., Böttcher S., Almeida J., van der Velden V.H.J., Flores-Montero J., Rawstron A., Asnafi V., Lécrevisse Q., Lucio P. (2012). EuroFlow antibody panels for standardized n-dimensional flow cytometric immunophenotyping of normal, reactive and malignant leukocytes. Leukemia.

[9] Rai K.R., Sawitsky A., Cronkite E.P., Chanana A.D., Levy R.N., Pasternack B.S. (1975). Clinical staging of chronic lymphocytic leukemia. Blood.

[10] Binet J.L., Auquier A., Dighiero G., Chastang C., Piguet H., Goasguen J., Vaugier G., Potron G., Colona P., Oberling F. (1981). A new prognostic classification of chronic lymphocytic leukemia derived from a multivariate survival analysis. Cancer.

[11] deAndrés-Galiana E.J., Fernández-Martínez J.L., Luaces O., del Coz J.J., Huergo-Zapico L., Acebes-Huerta A., González S., González-Rodríguez A.P. (2016). Analysis of clinical prognostic variables for Chronic Lymphocytic Leukemia decision-making problems. J. Biomed. Inform..

[12] Hampel P.J., Parikh S.A. (2022). Chronic lymphocytic leukemia treatment algorithm 2022. Blood Cancer J..

[13] Hallek M., Cheson B.D., Catovsky D., Caligaris-Cappio F., Dighiero G., Döhner H., Hillmen P., Keating M., Montserrat E., Chiorazzi N. (2018). iwCLL guidelines for diagnosis, indications for treatment, response assessment, and supportive management of CLL. Blood.

[14] Ten Hacken E., Gounari M., Ghia P., Burger J.A. (2019). The importance of B cell receptor isotypes and stereotypes in chronic lymphocytic leukemia. Leukemia.

[15] Hamblin T.J., Davis Z., Gardiner A., Oscier D.G., Stevenson F.K. (1999). Unmutated Ig V(H) genes are associated with a more aggressive form of chronic lymphocytic leukemia. Blood.

[16] Seifert M., Sellmann L., Bloehdorn J., Wein F., Stilgenbauer S., Dürig J., Küppers R. (2012). Cellular origin and pathophysiology of chronic lymphocytic leukemia. J. Exp. Med..

[17] Agathangelidis A., Chatzidimitriou A., Gemenetzi K., Giudicelli V., Karypidou M., Plevova K., Davis Z., Yan X.J., Jeromin S., Schneider C. (2021). Higher-order connections between stereotyped subsets: Implications for improved patient classification in CLL. Blood.

[18] Iyer P., Wang L. (2023). Emerging Therapies in CLL in the Era of Precision Medicine. Cancers.

[19] Agathangelidis A., Chatzidimitriou A., Chatzikonstantinou T., Tresoldi C., Davis Z., Giudicelli V., Kossida S., Belessi C., Rosenquist R., Ghia P. (2022). Immunoglobulin gene sequence analysis in chronic lymphocytic leukemia: The 2022 update of the recommendations by ERIC, the European Research Initiative on CLL. Leukemia.

[20] Galieni P., Troiani E., Picardi P., Angelini M., Mestichelli F., Dalsass A., Maravalle D., Camaioni E., Bigazzi C., Caraffa P. (2024). Unmutated IGHV at diagnosis in patients with early stage CLL independently predicts for shorter follow-up time to first treatment (TTFT). Leuk. Res..

[21] Jiménez de Oya N., De Giovanni M., Fioravanti J., Übelhart R., Di Lucia P., Fiocchi A., Iacovelli S., Efremov D.G., Caligaris-Cappio F., Jumaa H. (2017). Pathogen-specific B-cell receptors drive chronic lymphocytic leukemia by light-chain-dependent cross-reaction with autoantigens. EMBO Mol. Med..

[22] Del Giudice I., Chiaretti S., Santangelo S., Tavolaro S., Peragine N., Marinelli M., Ilari C., Raponi S., Messina M., Nanni M. (2014). Stereotyped subset #1 chronic lymphocytic leukemia: A direct link between B-cell receptor structure, function, and patients’ prognosis. Am. J. Hematol..

[23] Agathangelidis A., Chatzikonstantinou T., Stamatopoulos K. (2024). B-cell receptor immunoglobulin stereotypy in chronic lymphocytic leukemia: Key to understanding disease biology and stratifying patients. Semin. Hematol..

[24] Hallek M. (2019). Chronic lymphocytic leukemia: 2020 update on diagnosis, risk stratification and treatment. Am. J. Hematol..

[25] Agathangelidis A., Darzentas N., Hadzidimitriou A., Brochet X., Murray F., Yan X.J., Davis Z., van Gastel-Mol E.J., Tresoldi C., Chu C.C. (2012). Stereotyped B-cell receptors in one-third of chronic lymphocytic leukemia: A molecular classification with implications for targeted therapies. Blood.

[26] Stamatopoulos K., Belessi C., Moreno C., Boudjograh M., Guida G., Smilevska T., Belhoul L., Stella S., Stavroyianni N., Crespo M. (2007). Over 20% of patients with chronic lymphocytic leukemia carry stereotyped receptors: Pathogenetic implications and clinical correlations. Blood.

[27] von Elm E., Altman D.G., Egger M., Pocock S.J., Gotzsche P.C., Vandenbroucke J.P., Initiative S. (2007). Strengthening the Reporting of Observational Studies in Epidemiology (STROBE) statement: Guidelines for reporting observational studies. BMJ.

[28] Marinelli M., Ilari C., Xia Y., Del Giudice I., Cafforio L., Della Starza I., Raponi S., Mariglia P., Bonina S., Yu Z. (2016). Immunoglobulin gene rearrangements in Chinese and Italian patients with chronic lymphocytic leukemia. Oncotarget.

[29] Yao C.Y., Agathangelidis A., Chuang S.S., Tsou H.H., Feng W.L., Liu T.C., Chen T.Y., Yu Y.B., Yeh S.P., Yao M. (2022). Distinct Immunogenetic Profiles of Chronic Lymphocytic Leukemia in Asia: A Taiwan Cooperative Oncology Group Registry Study. Hemasphere.

[30] Wu S.J., Lin C.T., Agathangelidis A., Lin L.I., Kuo Y.Y., Tien H.F., Ghia P. (2017). Distinct molecular genetics of chronic lymphocytic leukemia in Taiwan: Clinical and pathogenetic implications. Haematologica.

[31] Huang Y.J., Kuo M.C., Chang H., Wang P.N., Wu J.H., Huang Y.M., Ma M.C., Tang T.C., Kuo C.Y., Shih L.Y. (2019). Distinct immunoglobulin heavy chain variable region gene repertoire and lower frequency of del(11q) in Taiwanese patients with chronic lymphocytic leukaemia. Br. J. Haematol..

[32] Baliakas P., Hadzidimitriou A., Sutton L.A., Minga E., Agathangelidis A., Nichelatti M., Tsanousa A., Scarfò L., Davis Z., Yan X.J. (2014). Clinical effect of stereotyped B-cell receptor immunoglobulins in chronic lymphocytic leukaemia: A retrospective multicentre study. Lancet Haematol..

[33] Bomben R., Dal Bo M., Capello D., Forconi F., Maffei R., Laurenti L., Rossi D., Del Principe M.I., Zucchetto A., Bertoni F. (2009). Molecular and clinical features of chronic lymphocytic leukaemia with stereotyped B cell receptors: Results from an Italian multicentre study. Br. J. Haematol..

[34] Ntoufa S., Papakonstantinou N., Apollonio B., Gounari M., Galigalidou C., Fonte E., Anagnostopoulos A., Belessi C., Muzio M., Ghia P. (2016). B Cell Anergy Modulated by TLR1/2 and the miR-17∼92 Cluster Underlies the Indolent Clinical Course of Chronic Lymphocytic Leukemia Stereotyped Subset #4. J. Immunol..

[35] Murray F., Darzentas N., Hadzidimitriou A., Tobin G., Boudjogra M., Scielzo C., Laoutaris N., Karlsson K., Baran-Marzsak F., Tsaftaris A. (2008). Stereotyped patterns of somatic hypermutation in subsets of patients with chronic lymphocytic leukemia: Implications for the role of antigen selection in leukemogenesis. Blood.

[36] Gaidano G., Rossi D. (2017). The mutational landscape of chronic lymphocytic leukemia and its impact on prognosis and treatment. Hematol. Am. Soc. Hematol. Educ. Program.

[37] Wierda W.G., Wang X., Obrien S., Faderl S., Ferrajoli A., Thomas D., Ravandi F., Cortes J., Giles F., Koller C. (2005). Serum Beta-2 Microglobulin: The Universal Independent Prognostic Factor for Patients with CLL. Blood.

[38] White A.M., Best O.G., Hotinski A.K., Kuss B.J., Thurgood L.A. (2023). The Role of Cholesterol in Chronic Lymphocytic Leukemia Development and Pathogenesis. Metabolites.

