# Supplementary material for: Analysis of Stereotyped B-Cell Receptor Frequencies Among Portuguese De Novo-Diagnosed Chronic Lymphocytic Leukemia Patients (PAIS Study)
Source: Cancers (Basel). 2025 Apr 14;17(8):1316. doi: 10.3390/cancers17081316 (PMC12025437; doi:10.3390/cancers17081316)
Supplement: Supplementary file 1 [file cancers-17-01316-s001.zip › cancers-3531407-supplementary.pdf]

Supplementary Data

Table S1. Distribution of the BCR stereotypes of newly diagnosed Portuguese CLL patients according to birthplace (n (%)).

Stereotype	#1	#2	#3	#4	#5	#6	#7H	#8	#12	#14	#16	#28A	#31	#59	#64B	#77	#99	#201	#202	Heterogeneous	Missing	Total
Aveiro	2 (2.3)		1 (1.2)	1 (1.2)		2 (2.3)		1 (1.2)		1 (1.2)	1 (1.2)					1 (1.2)				68 (79.1)	8 (8.3)	86
Beja	2 (7.7)																1 (3.8)			19 (73.1)	4 (15.4)	26
Braga	1 (4.3)					1 (4.3)														21 (91.3)		23
Bragança																				6 (100.0)		6
Castelo Branco																				10 (100.0)		10
Coimbra	1 (5.0)	1 (5.0)																		18 (90.0)		20
Évora				1 (14.3)																6 (85.7)		7
Faro				1 (9.1)				1 (9.1)												8 (72.7)	1 (9.1)	11
Guarda	1 (10.0)																			8 (80.0)	1 (10.0)	10
Leiria		1 (5.6)										1 (5.6)								15 (83.3)	1 (5.6)	18
Lisboa	6 (6.2)													2 (2.1)	1 (1.0)					81 (83.5)	7 (7.2)	97
Portalegre																				8 (100.0)		8
Porto	1 (2.4)				1 (2.4)															37 (90.2)	2 (4.9)	41
Santarém	1 (2.9)					1 (2.9)										2 (5.9)				29 (85.3)	1 (2.9)	34
Setúbal																				15 (93.8)	1 (6.3)	16
Viana do Castelo																						0

Vila Real								1 (9.1)										1 (9.1)		9 (81.8)		11
Viseu	2 (5.4)							1 (2.7)												33 (89.2)	1 (2.7)	37
Açores																				1 (100.0)		1
Madeira																				1 (100.0)		1
Total	17	2	1	3	1	4	0	4	0	1	1	1	0	2	1	3	1	1	0	393	27	463

Data are shown as *n* (%). Values in brackets indicate the percentage of the respective stereotype within the patient population of each district.

Table S2. Distribution of the *IGHV* mutational status of newly diagnosed Portuguese CLL patients according to birthplace.

Birthplace	Mutated *IGHV* (*n* = 265)	Unmutated *IGHV* (*n* = 171)
	***n* (%)**	***n* (%)**
**Aveiro (*n*=78)**	54 (69.2)	24 (30.8)
**Beja (*n*= 22)**	13 (59.1)	9 (40.9)
**Braga (*n*=23)**	14 (60.9)	9 (39.1)
**Bragança (*n*=6)**	3 (50.0)	3 (50.0)
**Castelo Branco (*n*=10)**	6 (60.0)	4 (40.0)
**Coimbra (*n*=20)**	15 (75.0)	5 (25.0)
**Évora (*n*=7)**	1 (14.3)	6 (85.7)
**Faro (*n*=10)**	5 (50.0)	5 (50.0)
**Guarda (*n*=9)**	6 (66.7)	3 (33.3)
**Leiria (*n*=17)**	11 (64.7)	6 (35.3)
**Lisbon (*n*=90)**	52 (57.8)	38 (42.2)
**Portalegre (*n*=8)**	6 (75.0)	2 (25.0)
**Oporto (*n*=39)**	25 (64.1)	14 (35.9)
**Santarém (=33)**	14 (42.4)	19 (57.6)
**Setúbal (*n*=15)**	10 (66.7)	5 (33.3)
**Viana do Castelo (*n*=0)**	0	0
**Vila Real (*n*=11)**	7 (63.6)	4 (36.4)
**Viseu (*n*=36)**	21 (58.3)	15 (41.7)
**Azores (*n*=1)**	1 (100.0)	0
**Madeira (*n*=1)**	1 (100.0)	0
**Missing**	0	0
